# One-pot biosynthesis of N-acetylneuraminic acid from chitin *via* combination of chitin-degrading enzymes, N-acetylglucosamine-2-epimerase, and N-neuraminic acid aldolase

**DOI:** 10.3389/fmicb.2023.1156924

**Published:** 2023-03-21

**Authors:** Quanzhen Liu, Guoguang Wei, Pengfan Yang, Chengyong Wang, Kequan Chen, Pingkai Ouyang, Alei Zhang

**Affiliations:** State Key Laboratory of Materials-Oriented Chemical Engineering, College of Biotechnology and Pharmaceutical Engineering, Nanjing Tech University, Nanjing, China

**Keywords:** N-Acetylneuraminic acid, N-acetyl-d-glucosamine, chitin, multi-enzyme catalysis, pyruvate

## Abstract

N-acetylneuraminic acid (Neu5Ac) possesses the ability to promote mental health and enhance immunity and is widely used in both medicine and food fields as a supplement. Enzymatic production of Neu5Ac using N-acetyl-D-glucosamine (GlcNAc) as substrate was significant. However, the high-cost GlcNAc limited its development. In this study, an *in vitro* multi-enzyme catalysis was built to produce Neu5Ac using affordable chitin as substrate. Firstly, exochitinase *Sm*ChiA from *Serratia proteamaculans* and N-acetylglucosaminosidase *Cm*NAGase from *Chitinolyticbacter meiyuanensis* SYBC-H1 were screened and combined to produce GlcNAc, effectively. Then, the chitinase was cascaded with N-acetylglucosamine-2-epimerase (AGE) and N-neuraminic acid aldolase (NanA) to produce Neu5Ac; the optimal conditions of the multi-enzyme catalysis system were 37°C and pH 8.5, the ratio of AGE to NanA (1:4) and addition of pyruvate (70 mM), respectively. Finally, 9.2 g/L Neu5Ac could be obtained from 20 g/L chitin within 24 h along with two supplementations with pyruvate. This work will lay a good foundation for the production of Neu5Ac from cheap chitin resources.

## 1. Introduction

N-acetylneuraminic acid (Neu5Ac), as one of the important members of the sialic acids family, plays an important role in regulating biological recognition, cellular immunity, and diseases (Tao et al., 2010). Among other research, Neu5Ac has proven to possess the function of promoting infant intelligence development and has been successively certified as an additive material (Zhang et al., 2019). In the medicine, Neu5Ac was used to embellish cell for targeted therapy of cancer, and to synthesize other structural drugs.

Currently, Neu5Ac can be prepared *via* natural extraction, chemical synthesis, microbial fermentation, and enzymatic catalysis. Extraction from natural substances such as milk, eggs, and cubilose is expensive because of the extremely low content of animal-derived resources and lots of complex operations (Koketsu, 1999). Chemical production of Neu5Ac requires harsh reaction conditions, expensive, and toxic metal catalysts, as well as environmental pollution (Furuhata, 2004). To improve production, microbial fermentation and enzymatic catalysis have been getting more attention and significant progress has been made in the production of Neu5Ac. By microbial fermentation using metabolically engineered microorganisms such as *Escherichia coli* (*E. coli*) DH5α, *Bacillus subtilis*, and *Serratia marcescens* as the production host, the enzyme related to Neu5Ac biosynthesis were overexpressed and three pathways have been built to produce Neu5Ac using different carbon sources such as glucose and glycerol (Honda et al., 1997; Lundgren and Boddy, 2007; Fierfort and Samain, 2008; Kang et al., 2012; Yan and Fong, 2018). Although many innovative engineering strategies have been developed and applied to the *de novo* synthesis of Neu5Ac, the current Neu5Ac titer of *de novo* synthesis is still low, causing difficulties in industrial applications. In enzymatic catalysis, N-acetyl-D-glucosamine as a substrate can be efficiently catalyzed into Neu5Ac *via* cascade catalysis of two enzymes: N-acetylglucosamine-2-epimerase (AGE) catalyzes the reversible reaction of N-acetyl-D-glucosamine (GlcNAc) to N-acetyl-D-manosamine (ManNAc), subsequently, N-acetyl-neuraminic acid aldolase (NanA) catalytic synthesis of Neu5Ac from ManNAc and pyruvate (Lee et al., 2004; Kao et al., 2018). Compared with biological fermentation the enzyme catalysis method has many advantages such as simplicity, mild catalytic conditions, and high conversion rates. However, the high cost of substrate GlcNAc limited the enzymatic production of Neu5Ac.

Chitin is a polymer consisting of the GlcNAc linked by a β-1,4-glycosidic bond, the second most abundant carbohydrate after cellulose, and which mainly derived from shrimp and crab shells (Tharanathan and Kittur, 2003; Shamshina et al., 2019). The GlcNAc can be obtained by hydrolysis of chitin (Cao et al., 2022). Among the hydrolysis method, acid hydrolysis is relatively efficient but results in low yields, low product quality, and risks associated with environmental pollution (Zhang et al., 2016; Le and Yang, 2019). Recently, enzymatic hydrolysis was developed for GlcNAc production, showing significantly enhance productivity and being environmentally friendly (Su et al., 2021). However, the highly crystalline structure of chitin due mainly to hydrogen bonding between chains hinders the process of enzymatic hydrolysis (Zhang et al., 2018b). To overcome this drawback, synergistic hydrolysis by multiple chitinases (endochitinase, exochitinase, and N-acetylglucosidase) has been investigated specifically to increase hydrolysis efficiency (Dahiya et al., 2006). In our previous study, we obtained many chitin-degrading enzymes from chitinase-producing bacteria *Chitinolyticbacter meiyuanensis* SYBC-H1, *Chitinolyticbacter* sp. GC72 and *Serratia proteamaculans* (Wei et al., 2017; Zhang et al., 2020a,b). The chitinases have been characterized that can efficiently convert chitin to GlcNAc (Zhang et al., 2018a; Chen et al., 2022; Wang et al., 2022). In this study, an *in vitro* multi-enzyme catalyzed system that the chitin was converted to Neu5Ac by one pot built. The system was optimized, and the production process of Neu5Ac was enhanced. This work will lay a good foundation for the production of Neu5Ac from cheap chitin resources.

## 2. Materials and methods

### 2.1. Chemicals

N-acetyl-D-glucosamine (GlcNAc), N-acetyl-D-manosamine (ManNAc) and N-acetylneuraminic acid (Neu5Ac) were purchased from Aladdin Reagent Co., Ltd. (Shanghai, China). Peptone and yeast extract were purchased from Oxoid Co., Ltd. (Beijing, China). All molecular reagents were purchased from TaKaRa (Dalian, China). Colloidal chitin was prepared as described by Gao et al. (2015). Other chemicals and solvents used in this study were purchased from local suppliers and were of analytical grade.

### 2.2. Plasmids and strains construction

*E. coli* Trans1-T1 was used for plasmid construction and propagation, and *E. coli* BL21(DE3) was used for protein expression. The expression vectors pET-32a (+) and pET-28a (+) were supplied by Novagen Co., Ltd. Plasmid construction and DNA manipulation were performed following the standard molecular cloning protocols. The gene of AGE (GenBank: ABG57043.1) obtained from *Anabaena* sp. CH1 was all codon-optimized and inserted into pET-32a (+). The genes of *Sm*ChiC (GenBank: ALE94877.1), *Sp*ChiA (GenBank: CP045913.1), *Cm*NAGase (GenBank: CP041335.1), NAGaseA and NanA (GenBank: CP025534.1) were amplified, respectively, from genomic DNA of *Serratia marcescens*, *Serratia proteamaculans* NJ303 (Wei et al., 2017), *Chitinolyticbacter meiyuanensis* SYBC-H1 (Zhang et al., 2020a), *Chitinolyticbacter sp.* GC72 and *Corynebacterium glutamicum* ATCC13032, and they were all codon-optimized and inserted into pET-28a(+). The plasmids, strains, primers, and specific structures of plasmids of using in this study are demonstrated in Supplementary Table S1.

### 2.3. Cell culture

Luria-Bertani (LB) medium was used for cell cultivation. All the single colonies of recombinant strains mentioned above were separately inoculated into 5 mL LB media containing kanamycin of 25 mg/L or ampicillin of 50 mg/L and incubated at 37°C and 200 rpm for 12 h. Then, the cultures (1 mL) were inoculated into 100 mL LB medium containing kanamycin of 25 mg/L or ampicillin of 50 mg/L in a 500 mL shake flask at 37°C with shaking at 200 rpm. When the OD_600_ reached 0.6–0.9, the recombinants were induced at 25°C with a final concentration of 0.1 mM isopropyl-β-D-thiogalactopyranoside (IPTG) for 12 h. The cells were harvested by centrifugation at 6000 g and 4°C for 10 min and washed twice with 0.9% saline solution.

### 2.4. Purification of recombinant age, NanA, *Cm*NAGase, and *Sm*ChiA

The harvested cells were suspended with buffer A (500 mM NaCl, 50 mM imidazole, 50 mM PBS, pH 7.4) and lysed by JY92-IIN ultrasonication (Ningbo Xinzhi Biotechnology, Ltd., Ningbo, China). After cell disruption, the cellular debris was removed by centrifugation at 13,000 g and 4°C for 30 min. The AGE, NanA, *Cm*NAGase, and *Sp*ChiA were purified using a protein purification system (AKTA Pure 150; GE Healthcare Co., Fairfield, United States) with a Ni-NTA column (His Trap™ FF 5 mL). The bound proteins were eluted with buffer B (500 mM NaCl, 250 mM imidazole, 50 mM PBS, pH 7.4). The eluted fractions were passed through an ultrafiltration tube of 10 kDa (Millipore, USA) to remove the imidazole with storage buffer (20 mM phosphate buffer, 150 mM NaCl, 5% (w/v) glycerol, pH 7.4) and concentrate the enzyme solution. The pure enzyme solutions were stored at −80°C for prior use. The SDS-PAGE analyzed purity of the AGE and NanA (Supplementary Figure S1).

### 2.5. Enzyme assay

The activity of AGE was assayed by measuring its ability to transform GlcNAc into ManNAc. 20 μL free AGE was added to 0.98 mL reaction system containing 100 mM GlcNAc, 2.5 mM ATP, 2 mM MgCl_2_, and 100 mM Tris–HCl (pH 7.5). NanA activity was assayed by measuring its ability to condense ManNAc and pyruvate into Neu5Ac. 20 μL free NanA was added to 0.98 mL containing reaction system 50 mM pyruvate, 50 mM ManNAc and 100 mM Tris–HCl (pH 7.5). All the reaction mixture in the 2 mL centrifuge tube were incubated at 37°C for 30 min. To stop the reaction, the reaction mixtures were terminated by boiling for 5 min. After centrifugation at 12, 000 g for 2 min and filtration through a 0.22 μm membrane, the concentrations of the substrate and the product were analyzed by high-performance liquid chromatography (HPLC, Agilent 1260 system). All tests were performed in triplicate and 1 unit of enzyme activity was defined as the amount of enzyme needed to produce 1 μmol of product per min at 37°C.

The chitinase activity was determined by DNS colorimetry (3,5-dinitrosalicylic acid), using colloidal chitin as substrate, and the amount of reducing sugar released by hydrolyzing chitin was determined. 0.48 mL sodium phosphate buffer (0.05 M, pH 7.0), 0.5 mL 1% colloidal chitin, and 0.02 mL chitinase solution were mixed and put into a water bath at 37°C for 30 min, after boiling for 5 min, the reaction was terminated. 1 mL DNS reagent was added and boiled for 5 min. The supernatant was cooled to room temperature. After centrifugation, the absorbance of the supernatant was measured at 540 nm, and the inactivated enzyme was used as blank control. The enzyme activity was defined as the amount of enzyme needed to convert the substrate colloidal chitin to produce 1 μmol reducing sugar per minute at 37°C, which was defined as one activity unit (U).

### 2.6. Screening of chitin degrading enzymes

To compare the activity of different chitin-degrading enzymes, 1 mL reaction mixtures contained 0.5 mL of 1% colloidal chitin, 200 μL of 100 U/L *Sp*ChiA or *Sm*ChiC and 300 μL 200 mM PBS (Na_2_HPO_4_-NaH_2_PO_4,_ pH 7.0), and incubated at 37°C for 2 h. For the combination of the chitin-degrading enzymes, 1 mL reaction mixtures contained 0.5 mL of 1% colloidal chitin, 100 U/L *Sp*ChiA and 100 U/L *Cm*NAGase or NAGaseA, and incubated at 37°C for 2 h. 100 U/L *Sm*ChiC and 100 U/L *Cm*NAGase or NAGaseA was added in the same catalysis system. At the end of the reaction, the reaction mixtures were terminated by boiling for 5 min. The concentration of GlcNAc was analyzed.

### 2.7. Multi-enzyme catalyzed chitin to synthesize Neu5Ac

To produce Neu5Ac, the two protocols were projected. The first protocol is that the reaction of the degradation of chitin and the synthesis of Neu5Ac has been carried on at the same time. 1 mL reaction mixtures contained 100 mM Tris–HCl buffer (pH 7.5), 20 g/L colloidal chitin, 100 mM pyruvate, 2.5 mM ATP, 2 mM MgCl_2_, 200 U/L AGE, 500 U/L μL NanA, 100 U/L *Sp*ChiA and 200 U/L CmNAGase, and incubated at 37°C for 12 h. The reaction mixtures were terminated by boiling for 5 min. After centrifugation, the concentration of Neu5Ac in the supernatant was measured by HPLC. The second protocol is that the chitinase was added first. When the reaction mixture was incubated at 37°C for 12 h, the reaction was stopped, and the AGE and NanA were added to produce Neu5Ac. Moreover, the reaction condition was at 37°C for 12 h. After centrifugation, the concentration of Neu5Ac in the supernatant was measured by HPLC.

### 2.8. Condition optimization of *in vitro* multi-enzyme for producing Neu5Ac

The synthesis of Neu5Ac from chitin by multi-enzyme was carried out in a 1 mL reaction system with four enzymes, substrate and coenzymes. The effects of varying temperatures (25°C, 30°C, 35°C, 40°C, 45°C, and 50°C), pH (6.0, 6.5, 7.0, 7.5, 8.0, 8.5, and 9.0), concentration of pyruvate (20 mM, 50 mM, 70 mM, 100 mM, 200 mM, 300 mM, 400 mM, and 500 mM), and ratio of AGE to NanA (1,1, 1:2, 1:3, 1:4, 1:5, and 1:6) on multi-enzyme catalysis were investigated. A one-pot biosynthesis strategy was employed in 1 mL 100 mM Tris–HCl buffer (pH 7.5), initially including 20 g/L colloidal chitin, 20 mM pyruvate, 2 mM MgCl_2_, 2.5 mM ATP, 200 U/L AGE, 200 U/L NanA, 100 U/L *Sp*ChiA, and 200 U/L *Cm*NAGase at 37°C for 12 h. After centrifugation, the concentration of Neu5Ac in the supernatant was measured by HPLC.

### 2.9. Analytical method

Concentrations of GlcNAc, ManNAc, Neu5Ac, and pyruvate were analyzed by HPLC, equipped with a Bio-Rad Aminex HPX-87H column (300 mm × 7.8 mm) using a refractive index detector. The mobile phase consisted of 5 mM H_2_SO_4_ at 0.5 mL/min, and the column temperature is 55°C.

## 3. Results and discussion

### 3.1. Screening and combination of chitinases

**Figure 1:**
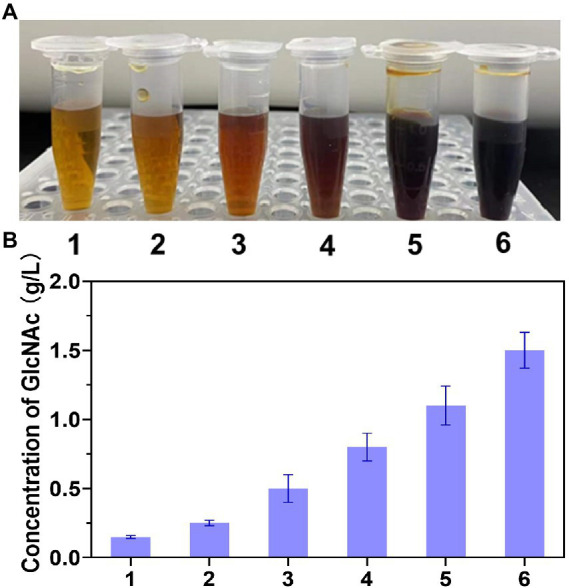
The degradation efficiency of different chitinase combinations. **(A)** Different reaction solutions treated by DNS colorimetry. **(B)** The concentration of GlcNAc under the degradation of different chitinase; (1: Chitinase *Sm*ChiC from *Serratia marcescens*; 2: Chitinase *Sp*ChiA from *proteamaculans* NJ303, 3: Combination of chitinase *Sm*ChiC and NAGasA from *Chitinolyticbacter* Sp. GC72, 4: Combination of chitinase *Sp*ChiA and NAGasA, 5: Combination of chitinase SmChiC and *Cm*NAGase from *Chitinolyticbacter meiyuanensis* SYBC-H1, 6: Combination of chitinase *Sp*ChiA and *Cm*NAGase).

Chitin-degrading enzymes, which are essential for chitin degradation, can be divided into endochitinase (casually cleaves chitin at internal sites to release N-acetyl chitooligosaccharides), exochitinase (hydrolyzes chitin to liberate GlcNAc dimer), and N-acetyl-β-glucosaminidase (converts N-acetyl chitooligosaccharides to GlcNAc) (Dahiya et al., 2006). To efficiently convert chitin to GlcNAc, a multienzyme system containing at least one chitinase and one N-acetyl-β-glucosaminidase (NAGase) is often required. Therefore, an exochitinase (*Sp*ChiA) from *Serratia proteamaculans* and an endochitinase (*Sm*ChiC) from *Serratia marcescens* combined with two N-acetyl-β-glucosaminosidase from *Chitinolyticbacter meiyuanensis* SYBC-H1 (*Cm*NAGase) and *Chitinolyticbacter* sp. GC72 (NAGaseA) respectively were chosen to hydrolyze chitin. As depicted in Figure 1A, the color of the combination of *Sp*ChiA and *Cm*NAGase was darkest, which demonstrated that the most reducing sugar was produced. In addition, the concentration of GlcNAc was analyzed. As demonstrated in Figure 1B, the combination of *Sp*ChiA and *Cm*NAGase could produce 1.4 g/L GlcNAc, which was higher than the production of *Sm*ChiC, *Sp*ChiA, the combination *Sp*ChiA and NAGaseA, the combination *Sm*ChiC and NAGaseA, and the combination of *Sp*ChiA and *Cm*NAGase. Moreover, the production of *Sp*ChiA and *Cm*NAGase co-catalysis was 5.6 times higher than the individual catalysis of *Sp*ChiA. Composite catalysis of exochitinase and N-acetyl-β-glucosaminosidase are effective for chitin hydrolysis to GlcNAc. The exochitinase can hydrolyze chitin to liberate GlcNAc dimer. The rapid accumulation of dimer inhibits the exochitinase activity. When the NAGaseA added in the system, the GlcNAc dimer was timely converted to GlcNAc and the inhibition was lifted. In the study performed by Zhu et al. (2016), three bacterial chitinases [chitinase A from *Serratia marcescens* (*Sm*ChiA), chitinase B from *Serratia marcescens* (*Sm*ChiB), and chitinase C from *Serratia marcescens* (*Sm*ChiC)] and one insect N-acetyl-D-glucosaminidase from *Ostrinia furnacalis* were combined to hydrolyze mycelial (contained chitin) producing GlcNAc, in which the higher hydrolytic activity was obtained after comparison with commercial chitinase. In our previous work, a novel multifunctional chitinase that has hydrolytic activity for chitin was characterized, and the activity of exochitinase and NAGase were demonstrated (Wang et al., 2022). Multi-enzyme synergy is an effective strategy for chitin hydrolysis. Therefore, this combination of *Sp*ChiA and *Cm*NAGase was chosen for chitin degradation.

### 3.2. Multi-enzyme cascade catalyzed synthesis of Neu5Ac

**SCHEME 1:**
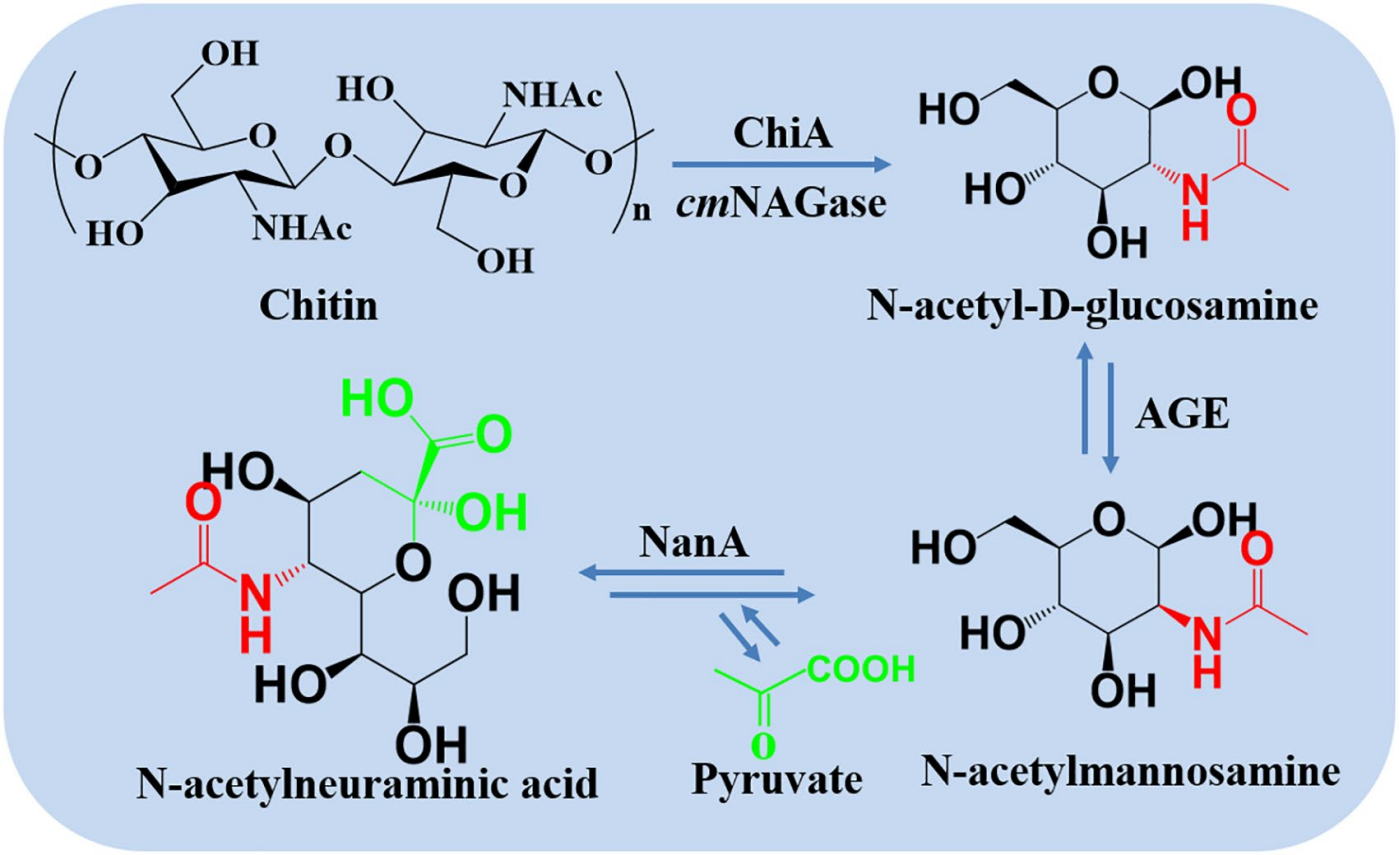
Scheme of Neu5Ac synthesis from chitin *via* an *in vitro* multi-enzyme.

The conversion process from chitin to Neu5Ac was demonstrated in Scheme 1, which is composed of the part consisting of chitin degradation and Neu5Ac synthesis, respectively. Two strategies such as one-pot and two-step catalysis were compared, and the results were demonstrated in Table 1; 6.1 g/L and 6.21 g/L of the Neu5Ac were synthesized by one-pot and two-step catalysis. Two processes had roughly the same catalytic efficiency. Thus, more efficient and time-saving one-pot catalysis was chosen for Neu5Ac production.

**Table 1:** The catalytic efficiency of two protocols.

Protocols	Neu5Ac (g/L)	Time (h)
One-pot	6.1 ± 0.02	12
Two-step	6.21 ± 0.13	24

### 3.3. Optimization of reaction conditions using *in vitro* multi-enzyme catalysis

**Figure 2:**
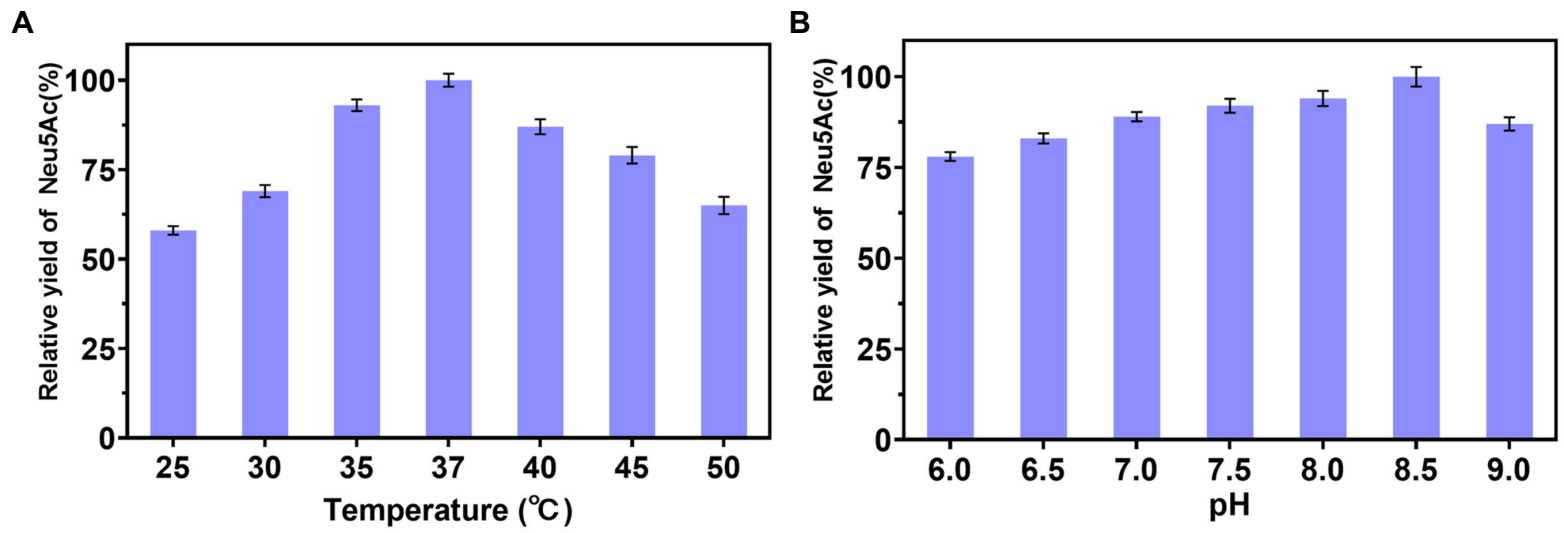
The effect of temperature and pH for the multi-enzyme cascade catalyzed. **(A)** Effect of temperature for the multi-enzyme cascade catalyzed synthesis. **(B)** Effects of pH for the multi-enzyme cascade catalyzed.

The temperature and pH were important influencing factors for enzyme catalysis. Therefore, the effects of temperature and pH on *in vitro* multi-enzyme catalysis were investigated. As depicted in Figure 2A, the relative yield of Neu5Ac increased as the reaction temperature increased from 25°C to 37°C, then decreased with the temperature increased from 37°C to 50°C. Thus, the optimal temperature was 37°C. As shown in Figure 2B, the relative yield of Neu5Ac increased with the increase in pH from 6.0 to 8.5 and further decreased at pH 9.0. A highest yield was obtained at pH 8.5. The result is similar to the optimum pH of NanA (Cheng et al., 2017). It could be that NanA is the rate-limiting step in Neu5Ac production. Therefore, the optimal catalytic conditions of the multi-enzyme cascade catalyzed was at 37°C and pH 8.5.

### 3.4. Effect of the ratio of age and NanA and pyruvate addition on multi-enzyme catalysis

In the bioconversion of Neu5Ac from GlcNAc, the synergy of AGE and NanA was important for the production of Neu5Ac, in which the activity of NanA was not only restrictive for the catalyze but also the activity of NanA was affected by the concentration of pyruvate (Daniels et al., 2014). Therefore, the effect of the ratio of AGE to NanA and the concentration of pyruvate were investigated. The effect of the concentration of enzyme on the production of Neu5Ac is demonstrated in Table 2. As the concentration of AGE increased, the production of Neu5Ac increased. When the ratio of AGE to NanA was 1:4, the highest 7.2 g/L of Neu5Ac was obtained, and the productivity was 0.6 g L^−1^h^−1^. Thus, the enzyme concentration of AGE 200 U/L and NanA 800 U/L was chosen as optimal in the following experiment.

**Table 2:** Effect of ratio of AGE to NanA on the yield and productivity of the multi-enzyme cascade catalyzed synthesis.

Enzymes activities (U/L)		Ratio of AGE to NanA	Neu5Ac (g/L)	Productivity (g L^−1^h^−1^)
AGE	NanA			
200	200	1:1	4.3	0.36
200	400	1:2	5.6	0.47
200	600	1:3	6.7	0.56
200	800	1:4	7.2	0.6
200	1,000	1:5	6.9	0.57
200	1,200	1:6	6.4	0.53

**Figure 3:**
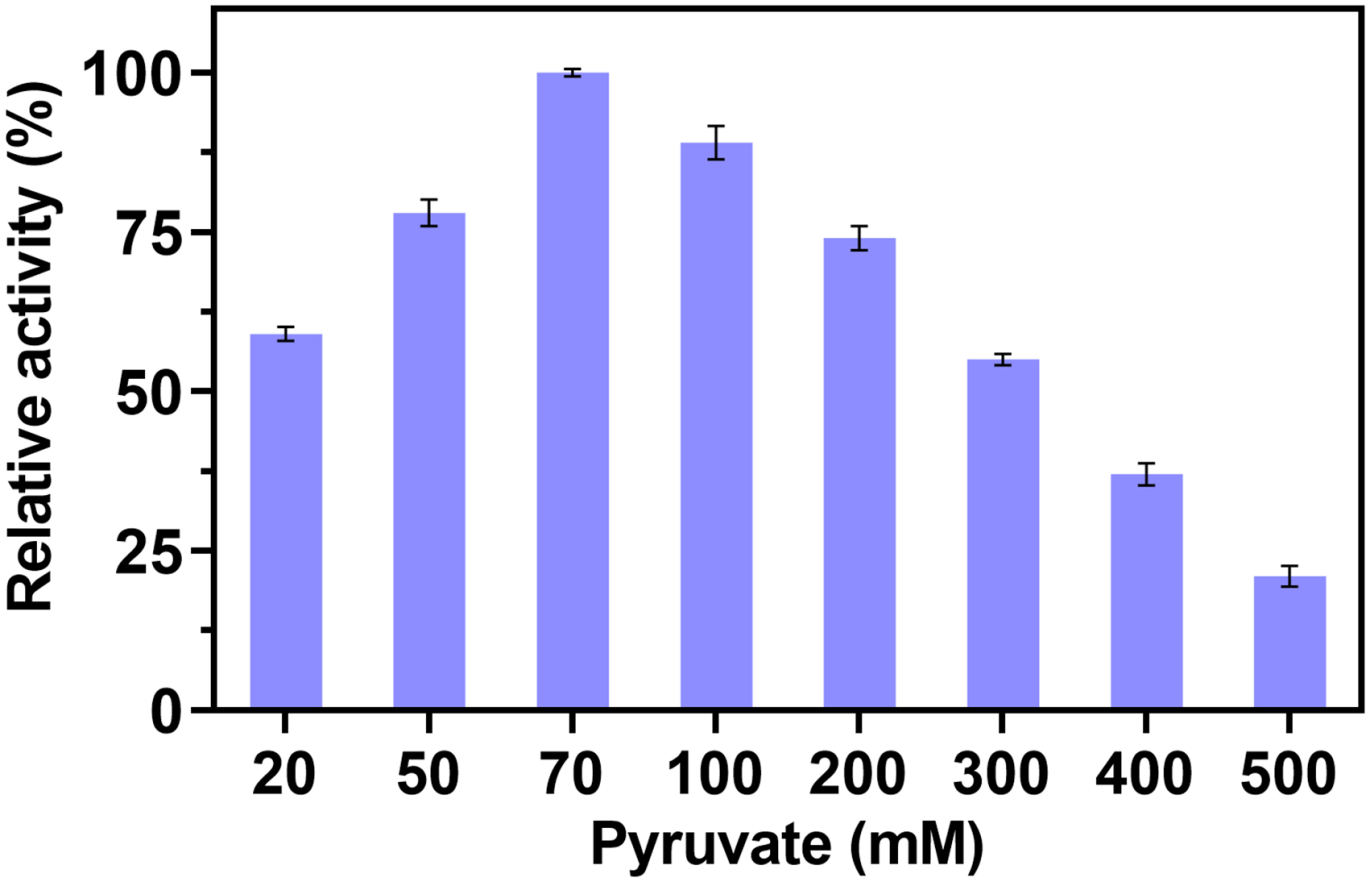
Effect of pyruvate concentration on the multi-enzyme catalyzed.

Based on the above-mentioned optimal conditions, the effect of concentration of pyruvate on the production of Neu5Ac was demonstrated in Figure 3. The relative activity increased with the concentration of pyruvate from 20 mM to 70 mM and reached maximum at 70 mM. However, when the concentration of pyruvate was improved continuously, the activity decreased gradually. The similar result was showed in Kao et al.’s (2018) study, by the optimization of concentration of GlcNAc and pyruvate, the high conversion rates of ManNAc were obtained. However, In Lee et al.’s (2004) study, the result showed that the higher ratio of pyruvate to GlcNAc can caused the problem of the enzyme inhibition. Their research showed that the N-acetylglucosamine-2-epimerase and N-neuraminic acid aldolase were inhibited by pyruvate. Therefore, the optimal concentration of pyruvate was 70 mM.

### 3.5. Synthesis of Neu5Ac in optimum conditions by *in vitro* multi-enzyme catalysis

**Figure 4:**
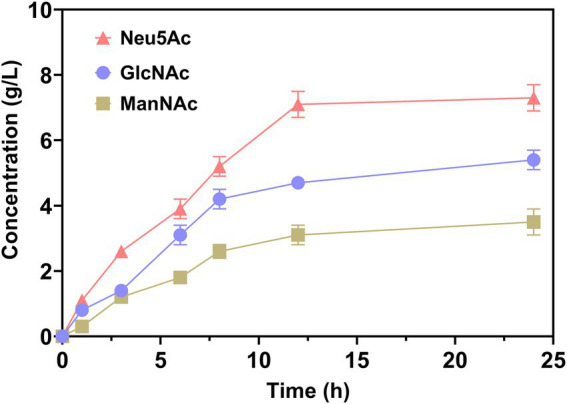
Neu5Ac production from chitin using enzyme cascade.

To evaluate the end and intermediate products in catalytic processes, the concentration of Neu5Ac, GlcNAc, and ManNAc was analyzed. As demonstrated in Figure 4, the concentrations of all mentioned above increased as the catalytic time increased. Within 12 h, the changes were increasing rapidly. Subsequently, the concentration increased slowly. The yield of Neu5Ac was approximately 7.3 g/L at 24 h. However, the intermediate products GlcNAc and ManNAc were approximately 5.1 and 2.4 g/L, respectively. It can be seen that accumulation of intermediate products was accumulated without being transformed into Neu5Ac. This is due to the reaction reaching equilibrium, since the catalysis of AGE and NanA was reversible (Maru et al., 2002; Wang et al., 2016).

### 3.6. Batch supplementation with pyruvate to improve Neu5Ac production

**Figure 5:**
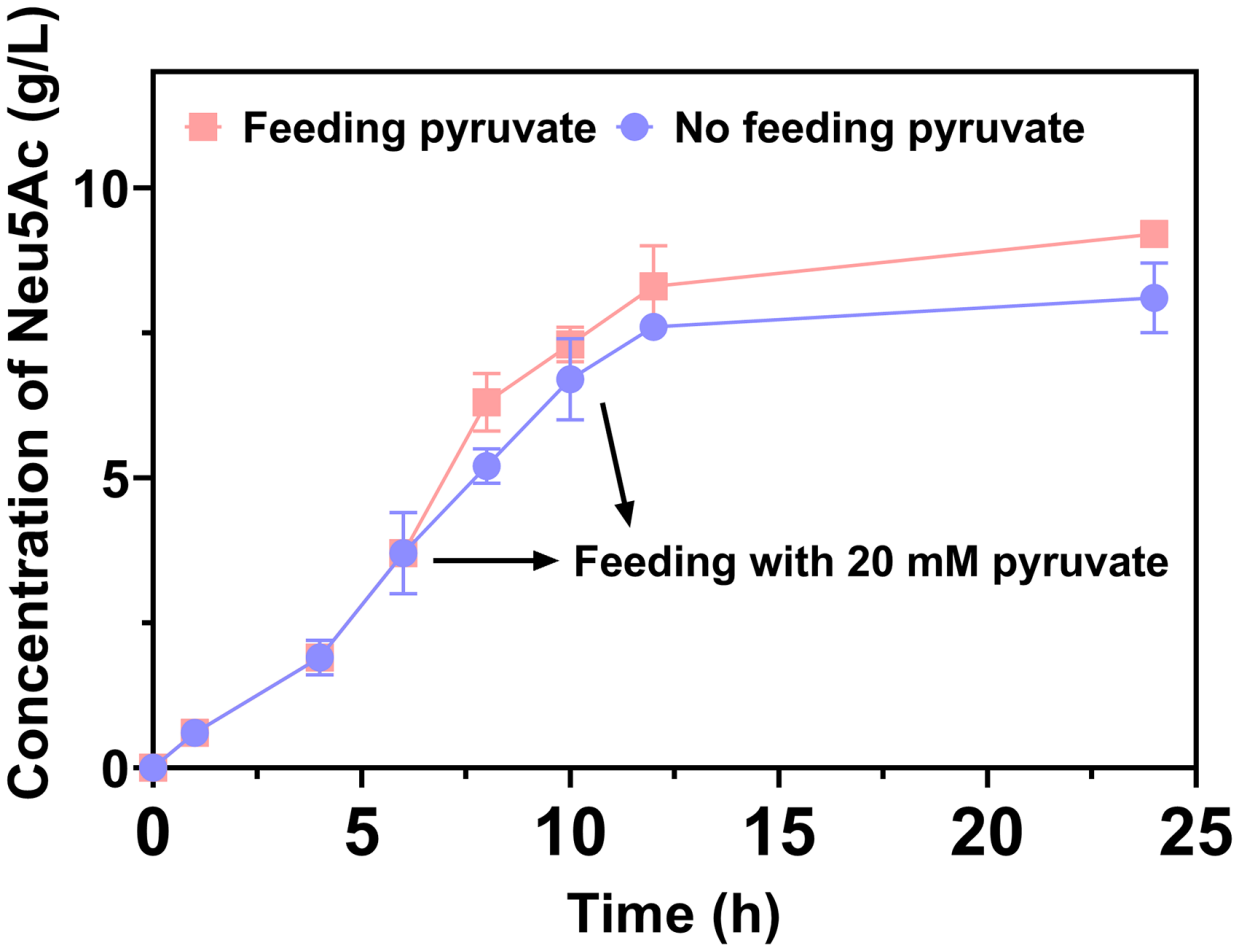
The production of Neu5Ac enhancement by feeding pyruvate.

In a study by Daniels et al. (2014), pyruvate was demonstrated as a co-substrate that was to be consumed. In the course of the reaction, the production of Neu5Ac was limited by the decrease in pyruvate. Thus, 20 mM of pyruvate was added at 6 h and 9 h, respectively, to increase the production of Neu5Ac. The result was demonstrated in Figure 5. The 9.2 g/L of Neu5Ac was obtained after catalytic 24 h. The addition of pyruvate usefully increased the production of Neu5Ac. In a study by Cheng et al. (2017), a similar result exhibited that the immobilized N-Acetylglucosamine-2-epimerase and N-acetylneuraminic acid lyase were used to produce Neu5Ac.

## 4. Conclusion

In this study, a system catalyzed by multi-enzyme cascade was constructed, in which chitinase was screened and compounded to hydrolyze chitin producing GlcNAc. Meanwhile, the GlcNAc was converted to Neu5Ac by combination of AGE and NanA. Based on Neu5Ac generation process optimization, fed-batch were adopted to enhance the bioconversion of chitin to Neu5Ac, in which the yield of Neu5Ac can reach 9.2 g/L. This report laid a good foundation for the biotransformation of Neu5Ac from chitin.

## Data availability statement

The original contributions presented in the study are included in the article/Supplementary material, further inquiries can be directed to the corresponding author.

## Author contributions

QL and GW wrote the original manuscript, conceived and designed the research, and analyzed the data. AZ, KC, and PO reviewed and edited the article. QL, GW, PY, and CW performed the experiments. All authors contributed to the article and approved the submitted version.

## Funding

This work was supported by the National Key Research and Development Program (no. 2021YFA0911400), National Natural Science Foundation of China (no. 22278220), the National Natural Science Foundation for Young Scientists of China (no. 21908101), the China Postdoctoral Science Foundation (no. 2022M710069), Jiangsu Agricultural Science and Technology Innovation Fund (CX (22) 3070), and the Jiangsu Province Excellent Postdoctoral Program (no. 2022ZB390).

## Conflict of interest

The authors declare that the research was conducted in the absence of any commercial or financial relationships that could be construed as a potential conflict of interest.

## Publisher’s note

All claims expressed in this article are solely those of the authors and do not necessarily represent those of their affiliated organizations, or those of the publisher, the editors and the reviewers. Any product that may be evaluated in this article, or claim that may be made by its manufacturer, is not guaranteed or endorsed by the publisher.
