# Supplementary material for: One-pot biosynthesis of N-acetylneuraminic acid from chitin via combination of chitin-degrading enzymes, N-acetylglucosamine-2-epimerase, and N-neuraminic acid aldolase
Source: Front Microbiol. 2023 Mar 21;14:1156924. doi: 10.3389/fmicb.2023.1156924 (PMC10072123; doi:10.3389/fmicb.2023.1156924)
Supplement: Supplementary file 1 [file Data_Sheet_1.docx]

Supplementary Material

One-pot biosynthesis of N-acetylneuraminic acid from chitin via combination of chitin-degrading enzymes, N-acetylglucosamine-2-epimerase, and N-neuraminic acid aldolase

**Quanzhen Liu^1 , †^, Guoguang Wei ^1, †^, Pengfan Yang^1^, Chengyong Wang^1^, Kequan Chen^1^, Pingkai Ouyang^1^, Alei Zhang^1,^***

*** Correspondence: Alei Zhang：zhangalei@njtech.edu.cn**

Table S1 List of strains and plasmids used for the production of Neu5Ac

| **Strains** | **Characteristics** | **Source** |
| --- | --- | --- |
| *E. coli* BL21(DE3) | Protein expression host | Novagen |
| *E. coli* Trans 1T1 | Cloning host | Novagen |
| *Chitinolyticbacter meiyuanensis* SYBC-H1 | Wild type, isolated from soil | This lab |
| *Corynebacterium glutamicum* ATCC13032 | - | This lab |
| **Plasmids** |  |  |
| pET-28a (+) | *E. coli* cloning vector; Kan^r^ | Novagen |
| pET-32a (+) | *E. coli* cloning vector; Amp^r^ | Novagen |
| pET-28a (+)-*SmChiA* | pET-28a (+), carrying the *Cm*NAGase gene from *Serratia proteamaculans* | This study |
| pET-28a (+)-*CmNAGase* | pET-28a (+), carrying the *Cm*NAGase gene from *Chitinolyticbacter meiyuanensis* SYBC-H1 | This study |
| pET-28a (+)-*NanA* | pET-28a(+), carrying the NanA gene from *Corynebacterium glutamicum* ATCC13032 | This study |
| pET-32a (+)-*AGE* | pET-32a(+), carrying the AGE gene from *Anabaena sp.* CH1 | This study |
| **Primers** |  |  |
| *SmChiA*-F-*BamH*I | GGTCGCGGATCCATGCGCAAATTTAATAAACCGCTG | - |
| *SmChiA*-R-*Hind*III | GCCGCAAGCTTTTGAACGCCGGCGCT | - |
| *CmNAGase*-F-*BamH*I | GGTCGCGGATCCATGAGCCGTCCCGCC | - |
| *CmNAGase*-R-*Hind*III | GCCGCAAGCTTGGCGCCCACCTGCA | - |
| *NanA*-F-*BamH*I | GGTCGCGGATCCATGGCTTCCGCAACTTTCACC | - |
| *NanA*-R-*Hind*III | GCCGCAAGCTTTTAAGCGGTGTACAGGAATTCATCAAC | - |
| *AGE*-F-*Hind*III | GGCCGCAAGCTTATGGGCAAAAATCTGCAGGC | - |
| *AGE*-R-*BamH*I | GATATCGGATCCGCTCAGTGCTTCAAACTGCTG | - |

*^a^*Underlined sequences within the primers are restriction sites. Amp^r^, ampicillin resistance; Kan^r^, kanamycin resistance.


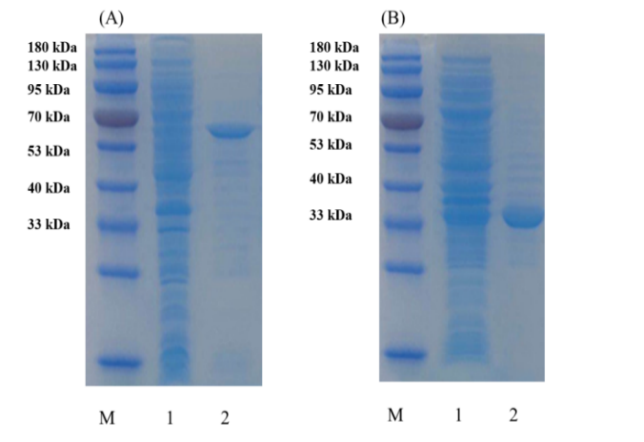


Figuer.S1 The SDS-PAGE analysis of enzyme AGE (A) and NanA (B)

- M：Maker, 1: Crude enzyme, 2: purified enzyme;
